# Potential of artificial intelligence based on chest computed tomography to predict the nature of part‐solid nodules

**DOI:** 10.1111/crj.13597

**Published:** 2023-02-05

**Authors:** Xiaoting Ke, Weiyi Hu, Xianyan Su, Fang Huang, Qingquan Lai

**Affiliations:** ^1^ Department of CT/MRI The Second Affiliated Hospital of Fujian Medical University Quanzhou China

**Keywords:** artificial intelligence, chest computed tomography, malignancy, part‐solid nodule

## Abstract

**Background:**

The potential of artificial intelligence (AI) to predict the nature of part‐solid nodules based on chest computed tomography (CT) is still under exploration.

**Objective:**

To determine the potential of AI to predict the nature of part‐solid nodules.

**Methods:**

Two hundred twenty‐three patients diagnosed with part‐solid nodules (241) by chest CT were retrospectively collected that were divided into benign group (104) and malignant group (137). Intraclass correlation coefficient (ICC) was used to assess the agreement in predicting malignancy, and the predictive effectiveness was compared between AI and senior radiologists. The parameters measured by AI and the size of solid components measured by senior radiologists were compared between two groups. Receiver operating characteristic (ROC) curve was chosen for calculating the Youden index of each quantitative parameter, which has statistical significance between two groups. Binary logistic regression performed on the significant indicators to suggest predictors of malignancy.

**Results:**

AI was in moderate agreement with senior radiologists (ICC = 0.686). The sensitivity, specificity and accuracy of two groups were close (*p* > 0.05). The longest diameter, volume and mean CT attenuation value and the largest diameter of solid components between benign and malignant groups were different significantly (*p* < 0.001). Logistic regression analysis showed that the longest diameter and mean CT attenuation value and the largest diameter of solid components were indicators for malignant part‐solid nodules, the threshold of which were 9.45 mm, 425.0 HU and 3.45 mm, respectively.

**Conclusion:**

Potential of quantitative parameter measured by AI to predict malignant part‐solid nodules can provide a certain value for the clinical management.

## Graphical abstract

In this work, the accuracy of using artificial intelligence (AI) to predict the nature of part‐solid nodules was reliable and comparable to that of senior radiologists. Combined with previous studies, the authors preliminarily believe that the qualitative determination of part‐solid pulmonary nodules by AI can provide a certain value for the clinical management of patients in their region.

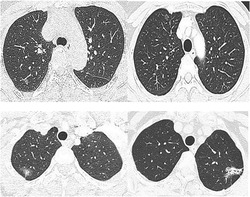

## 1 INTRODUCTION

Part‐solid nodules belong to the subsolid nodule (SSN) category, which means that they contain additional solid components of pulmonary nodules that can be observed in the lung or mediastinal window, excluding normal bronchi and vessels.^1^, ^2^ Most part‐solid nodules, except for those that disappear, tend to indicate invasive adenocarcinomas (IA), minimally invasive adenocarcinoma (MIA), adenocarcinomas in situ (AIS) and atypical adenomatous hyperplasia (AAH) to some extent.

Some researchers have explored the correlation between the pathological features of part‐solid nodules and the findings from chest computed tomography (CT) scans, which have contributed to the prediction of histological subtypes of adenocarcinoma.^3^, ^4^, ^5^ In terms of qualitative factors, Wang et al. noted that the risk of canceration for part‐solid nodules in Asia was also seen in non‐elderly or non‐smoking women.^6^, ^7^, ^8^, ^9^, ^10^ As for quantitative indicators, the solid component, associated CT value and volume are beginning to be followed with interest, in addition to the familiar size parameter.^11^, ^12^ The 2017 Fleischner guidelines and the American College of Radiology Lung CT Screening Reporting and Data System proposed different management recommendations for different sizes of entire nodules and solid components.^3^ Although several studies in recent years have suggested that mediastinal windows are suitable for the measurement of solid components,^3^, ^13^ lung windows with a high‐altitude frequency algorithm were strongly recommended by the revised guidelines published in 2017 for measuring solid components.^1^ Choi et al. have suggested that the lung window setting may be superior to the mediastinal window for the measurement of invasive components. This approach has considerable interobserver consistency and moderate accuracy in the prediction of pathological invasive components.^14^ Wu et al. demonstrated similar diagnostic performance between the mediastinal and lung windows for the solid component.^15^

To date, the research on computer‐aided diagnostic (CAD) systems for detection of pulmonary nodules has entered a mature stage, but their use for diagnosis is still exploratory. Some researchers have proposed the use of CAD to predict benign and malignant pulmonary nodules based on chest CT images, including classical machine learning methods and deep learning models.^15^, ^16^ Diversified feature extraction protocols were used in classical machine learning method to describe the features of nodules. Nevertheless, due to the multiple locations, sizes, shapes and densities of nodules, it was difficult to select suitable features for detection.^17^ The deep learning tool can automatically acquire features to detect and classify nodules. The method based on 3D convolution neural networks (CNN) makes the best of the information from sagittal, coronal and axial angles to improve the stability and credibility of diagnostic results.^18^ On the strength of previous findings, the diagnostic efficacy of the CNN model has been judged superior to the traditional CAD scheme. Actually, the automatic diagnosis algorithm based on 3D CNN, after continuous improvement, can be better than radiologists in judging the nature of pulmonary nodules. Gong et al. have proposed a CNN model based on deep learning to predict the possibility of ground glass nodules being adenocarcinoma.^19^ The study concluded that AI was better than radiologists at predicting aggressive adenocarcinomas. Due to the limitations of experience and the influence of workload, radiologists are prone to biased results. AI, because it is repeatedly improved and verified, is more objective and efficient than radiologists. However, the diagnostic performance of AI on benign and malignant pulmonary nodules still needs to be further explored and proved. Hence, this study aimed to explore the potential of AI based on 3D CNN to identify malignant part‐solid nodules based on chest CT.

## 2 MATERIALS AND METHODS

### 2.1 Patient population

The institutional review board of our hospital approved this retrospective study, and written informed consent was obtained. A search for patients with pulmonary SSNs on chest CT from September 2018 to January 2021 was conducted in the hospital's picture archiving and communication and radiology information system (PACS). Inclusion criteria were the following: (i) All pulmonary part‐solid nodules were excluded from false‐positive and false‐negative nodules, (ii) 3 mm < diameter of whole nodule ≤30 mm, (iii) CT image thickness of 1 or 1.25 mm and (iv) diagnostic reports included the terms ‘SSN’ or ‘part‐solid nodules’. Exclusion criteria were the following: (i) pulmonary diffuse lesions and (ii) serious artefacts in the image. Finally, 223 patients with 241 part‐solid nodules were brought into this retrospective study.

### 2.2 CT scanning

Two hundred twenty‐three patients were scanned by a 64‐row (Light Speed VCT, Discovery HDCT, Optima) spiral CT‐scanning machine from the entrance of the chest to the basis pulmonis. The patient completed the whole‐lung scan with one breath held after inhalation. Scanning mode: spiral scanning; tube current: 200–340 mA; screw pitch: 1.375:1; layer thickness: 5.0 mm; image matrix: 512, 512; field of view (FOV): 360 mm. The standard algorithm was used to reconstruct the 1.25‐or 1.0‐mm thick axial image.

### 2.3 AI software

The AI software provided by Beijing Yizhun Intelligent Technology Co. Ltd. (Chest CT Auto Diagnostic System V6.0), which was clinically proven and commercially available. The software is based on 3D CNN method to fully extract coronal, sagittal and transverse features. The image is transferred to AI software for automatic identification, labeling and completion the report.

### 2.4 Imaging processing and analysis

In the first stage, two senior radiologists (working more than 15 years) blind to pathology results simultaneously interpreted and analysed the characteristics of 241 nodules independently through morphologic changes and some quantitative indicators (size and density) by manual measurement without using AI software. The prediction results were recorded. When there are two different opinions in the group, a consensus is reached after discussion. In addition, the chest CT images of 241 nodules were imported into the AI system to obtain preliminary judgement results.

The thin‐slice lung windows of the 241 nodules confirmed by postoperative pathology were imported into the AI system to obtain the relevant quantitative parameters: the longest and shortest diameters of the whole nodules, the volume and the mean CT attenuation value. The longest and shortest diameters of the whole nodules were acquired in three‐dimensional space. In addition, the size of the solid component of each part‐solid nodule that was defined as the maximum diameter in the axial section on the lung window setting [window width, 1500 Hounsfield units (HU); level, −700 HU], was repeatedly measured three times by a radiologist with more than 10 years' experience and was taken as the average.

### 2.5 Statistical analysis

The SPSS 22.0 software (IBM Corp.) was chosen for statistical analysis. Intraclass correlation coefficient (ICC) was used to assess the agreement in predicting malignant part‐solid pulmonary nodules between AI and senior radiologists, with ICC value of <0.5, 0.5 to 0.75, 0.75 to 0.9 and >0.9 indicating poor, moderate, good and perfect agreement, respectively.^20^ McNemar's test was used to compare the sensitivity, specificity and accuracy between AI and senior radiologists in predicting the malignancy risk. Then qualitative indicators (gender, smoking or not) and quantitative indicators (the longest and shortest diameters of the whole nodules, the volume and the mean CT attenuation value and long diameter of solid component) measured by AI of benign and malignant groups were compared by chi‐square test and *t*‐test, respectively. All continuous variables are normally distributed, and the variables are expressed as mean ± standard deviation. The receiver operating characteristic (ROC) curve was chosen to calculate the area under the curve (AUC) and the Youden index of each quantitative parameter, which have statistical significance between two groups. In addition, binary logistic regression was applied to explore the predictors for malignant part‐solid nodules. *p < 0.05* was judged to indicate a significant difference.

## 3 RESULTS

### 3.1 Clinical characterization of study population

**FIGURE 1:**
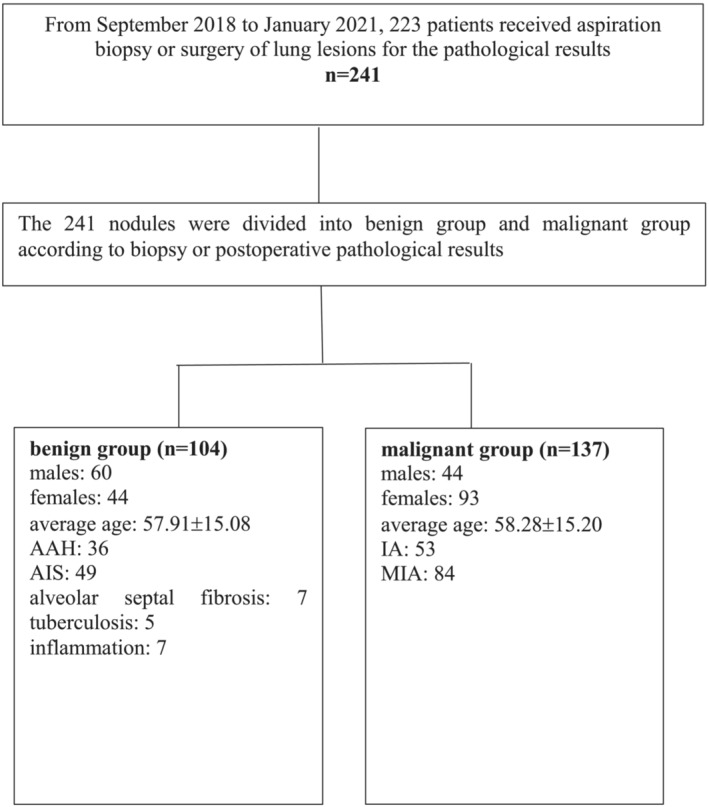
Study population

**FIGURE 2:**
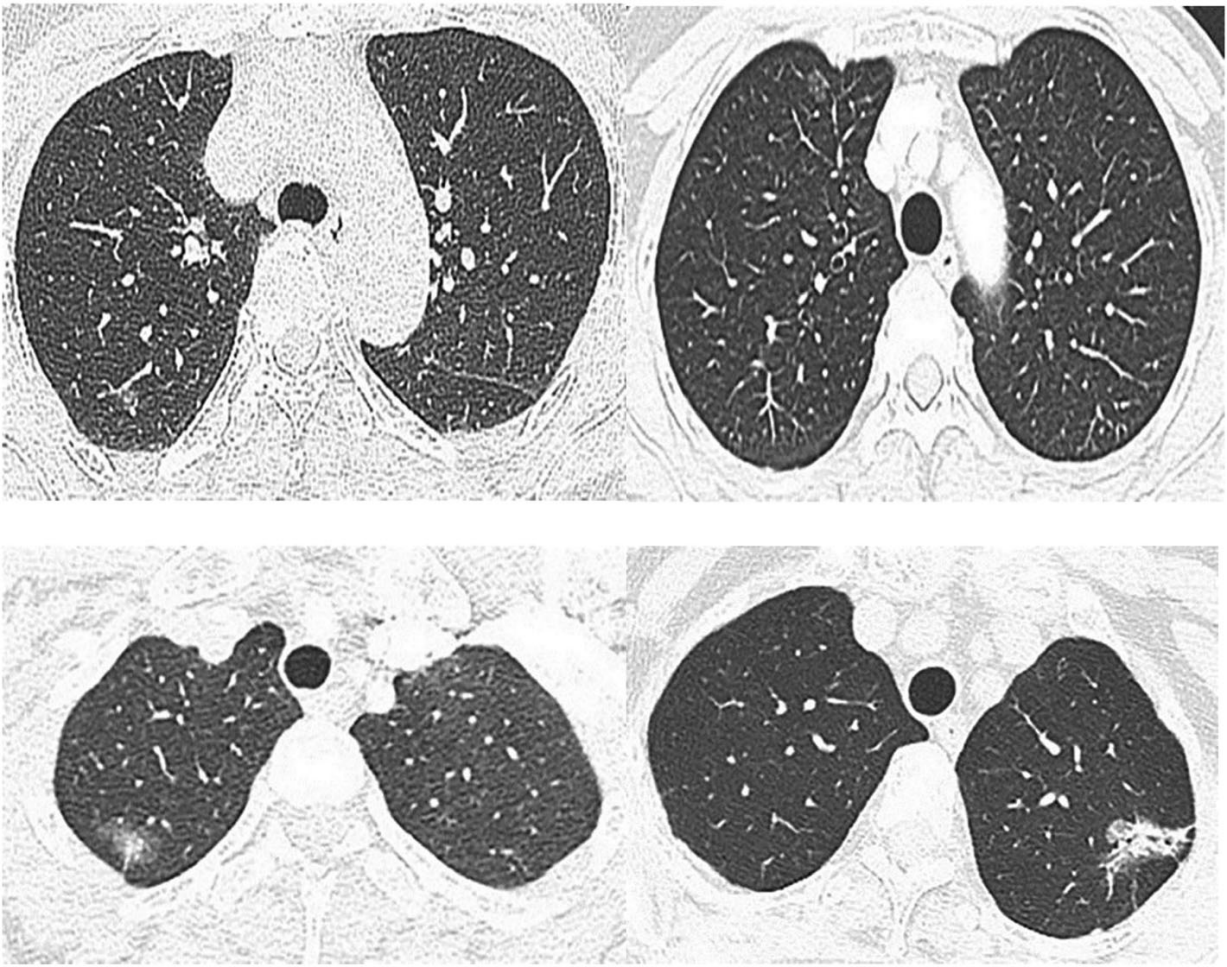
Part‐solid nodules at different pathological stages. (A) An atypical adenomatous hyperplasia (AAH) in the posterior segment of the right upper lobe. (B) An adenocarcinomas in situ (AIS) in the anterior superior lobe of the right lung. (C) A minimally invasive adenocarcinoma (MIA) in the apex segment of the right upper lobe. (D) An invasive adenocarcinomas (IA) in the upper lobe of the left lung

The 241 nodules were divided into benign group (104) and malignant group (137) according to biopsy or postoperative pathological results. Among the 104 benign part‐solid nodules, there were 60 males and 44 females, with an average age of 57.91 ± 15.08 years old, including 36 cases of AAH, 49 cases of AIS, seven cases of alveolar septal fibrosis, five cases of tuberculosis and seven cases of inflammation. Among the 137 malignant lesions, there were 44 males and 93 females, with an average age of 58.28 ± 15.20 years, including 53 cases of IA and 84 cases of MIA (Figures 1 and 2).

### 3.2 Predictive efficacy analysis of AI

Of the 241 part‐solid nodules, senior radiologists predicted 143 malignant nodules and 98 benign nodules. Senior radiologists misdiagnosed 6 true malignant nodules as benign nodules and 12 true benign nodules as malignant nodules. AI predicted 148 malignant nodules and 93 benign nodules. AI misdiagnosed 13 true malignant nodules as benign nodules and 24 true benign nodules as malignant nodules. In terms of the predictive malignancy, AI was in moderate agreement with senior radiologists (ICC = 0.686, 95% CI 0.613 to 0.747). See Table 1 for sensitivity, specificity and accuracy of two groups. However, the difference of sensitivity, specificity and accuracy between the two groups was not significant (*p* > 0.05).

**TABLE 1:** Comparison on the prediction efficiency for malignant part‐solid nodules between AI and senior radiologists

Variables	Senior radiologist	AI
True positive	131	124
False positive	12	24
False negative	6	13
True negative	92	80
Sensitivity	95.6% (131/137)	90.5% (124/137)
Specificity	88.5% (92/104)	76.9% (80/104)
Accuracy	92.5% (223/241)	84.6% (204/241)

### 3.3 Comparison of clinical characteristics, solid components and AI quantitative parameters between benign and malignant patients with part‐solid nodules based on chest CT

There were no significant differences in age, gender, smoking and the shortest diameter between benign and malignant groups (*p* > 0.05). The longest diameter of the whole nodule, volume, the mean CT attenuation value and the size of the solid component were 6.64 ± 1.90 mm, 301.0 ± 80.19 mm^3^, −509.93 ± 97.0 HU, 2.32 ± 0.66 mm in benign group and 10.61 ± 3.20 mm, 432.31 ± 154.70 mm^3^, −334.03 ± 139.46 HU, 4.48 ± 1.48 mm in malignant group. The largest diameter of the whole nodule, volume, mean CT attenuation value and size of solid component in the benign group were all less than those in the malignant group (*p* < 0.05, Table 2).

**TABLE 2:** Comparison of clinical characteristics and quantitative parameters between benign and malignant groups

Parameters	Benign group	Malignant group	T/χ^2^ value	*p*‐Value
Age (year)	57.91 ± 15.08	58.10 ± 15.20	0.096	0.608
Smoking/non‐smoking	61/43	51/86	0.079	0.453
Male/female	60/44	44/93	2.649	0.104
The longest diameter (mm)	6.64 ± 1.90	10.61 ± 3.20	11.22	<0.001
The shortest diameter (mm)	3.8 ± 0.827	4.1 ± 0.929	2.57	0.051
Volume (mm^3^)	301.0 ± 80.19	432.31 ± 154.70	7.86	<0.001
Mean CT attenuation value (HU)	−509.93 ± 97.0	−334.03 ± 139.46	10.98	<0.001
Solid component (mm)	2.32 ± 0.66	4.88 ± 1.48	13.78	<0.001

### 3.4 Analysis of diagnostic performance

**FIGURE 3:**
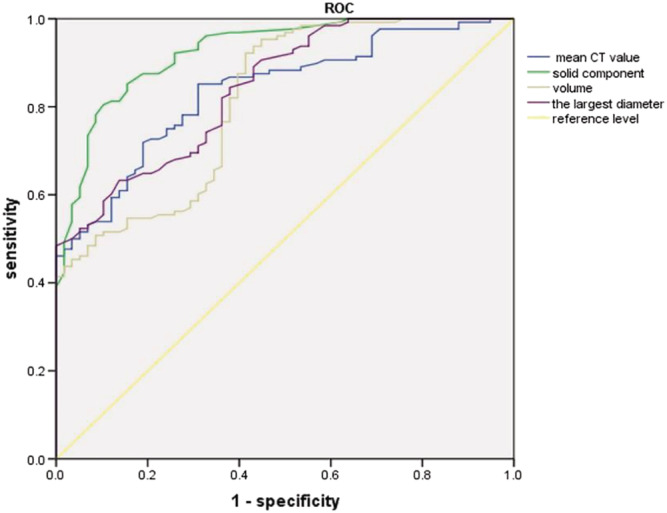
Receiver operator characteristic curve of quantitative parameters for part‐solid nodules

ROC curve was applied to analyse each parameter with significant difference between benign and malignant groups, and the corresponding Youden index was calculated (Figure 3). The AUC values of the longest diameter, mean CT attenuation value and volume and largest diameter of the solid component were 0.855, 0.843, 0.738 and 0.918, respectively. The threshold of the longest diameter was 9.45 mm, the threshold of the average CT attenuation value was 425.0 HU and the threshold of the largest diameter of the solid component was 3.45 mm. The sensitivity and specificity of the longest diameter, mean CT attenuation value and the largest diameter of solid component were 0.613 and 0.904, 0.730 and 0.808, 0.745 and 0.942. With the area under the curve (AUC) > 0.7 as the independent variable, the pathological findings were the dependent variables. Binary logistic regression analysis suggested that the longest diameter [odds ratio (OR) = 0.766, *p* = 0.011], mean CT attenuation value (OR = 0.993, *p* = 0.004) and solid component (OR = 0.191, *p* < 0.001) were independent impact indicators for a part‐solid nodule to be malignant.

## 4 DISCUSSION

In our study, we have revealed similar potential for the prediction of malignant risk of part‐solid nodules between AI and senior radiologists. The senior radiologist mainly depended on the morphological characterization, size and density of the nodules to determine the nature. AI determined benign or malignant lung nodules by quantitative parameters. By the ROC curve and the Youden index, we got the threshold values of the quantification parameters of malignancy that had significant difference in the comparison between benign group and malignant group and the corresponding sensitivities and specificities. Besides, this study considered that the longest diameter of the whole nodule, CT attenuation value and the solid component were the indicators of the malignancy of part‐solid nodules through binary logistic regression. Hence, we suggested that the quantitative parameters of part‐solid nodules measured by AI to predict the malignancy were valuable.

Lung cancer, with its high fatality rate, is one of the most common cancers in Asia, but some researchers have suggested that the 5‐year disease‐free survival after surgery of AIS and MIA is promising, even close to 100%, in patients with stage IA.^18^, ^21^ Hence, early detection, early diagnosis and early intervention are particularly pivotal. However, the indolent development of most part‐solid pulmonary nodules poses quite a challenge for clinicians to make rational decisions based on chest imaging information. Based on the current medical background, it is critical to effectively and timely identify the malignant part‐solid nodules by AI. Our study has explored the ability of AI to distinguish benign from malignant part‐solid pulmonary nodules and attempted to discover the clinical value of the quantitative index measured by AI.

To the authors' best knowledge, the distinction between benign and malignant SSNs has been based primarily on the overall size of the nodule, the size of the solid component and the experience of radiologists in recent clinical practice.^22^ If the imaging characteristics of part‐solid nodules were measured only by hand, the process would be tedious and time‐consuming. The application of AI can make up for these shortcomings. It can directly extract the nodule and quantify its relevant indicators to reduce manual measurement error. Encouragingly, the efficacy of AI in differentiating benign and malignant part‐solid pulmonary nodules in our study was close to that of senior radiologists.

Quantitative analysis of the structure of part‐solid pulmonary nodules has been considered as an approach to distinguish IA from preinvasive lesions in the recent research context. The quantitative indicators in this study included the longest diameter of whole nodules, the mean CT attenuation value, the volume, read by AI, and the solid component measured repeatedly by an experienced radiologist. In this study, the differences in the longest diameter, the mean CT attenuation value, the volume and the solid component between benign and malignant part‐solid nodules were statistically significant, which was consistent with the research of Kim et al.^2^, ^11^, ^23^, ^24^ Binary regression analysis indicated that the longest diameter, the mean CT value and the size of the solid component were independent impact factors in this research, apart from volume.

Revel et al. demonstrated in previous studies that the longest diameter of the whole nodule in a lung window setting can help to predict the invasiveness of SSNs.^3^ Similarly, the longest diameter of the whole nodule in this study was one of the independent impact factors. It was found that when its critical value was 9.45 mm, its sensitivity and specificity were 0.633 and 0.862, respectively. A previous analysis by Revel et al. based on the longest diameter between IA and preinvasive lesions suggested that the threshold was 10 mm measured manually.^3^ In addition, this study demonstrated that the mean CT value plays an important role in predicting malignant part‐solid nodules. Likewise, Borczuk et al. believed that the severity of pulmonary SSNs could be determined based on changes in size and density and that the deeper the invasion, the greater were the density and size.^23^, ^24^ Taking this further, Li et al. attempted to propose critical values for the mean and maximum CT attenuation values between AAH/AIS and MIA of −548.00 and −419.74 HU.^11^ The present study has found that the threshold of the mean CT attenuation value between AHH/AIS and MIA/IA was −427.5 HU. The difference in volume between benign and malignant groups was confirmed to be significant in the present case, but it was not considered as an impact factor in the binary regression analysis. To the authors' knowledge, the issue of how to ensure the accuracy of volume measurement is largely influenced by the segmentation algorithm (or software), the CT acquisition protocol and the characteristics of the nodule (such as density or location).^2^ Although the volumetric measurement has the advantage of integrating the overall solid contour of a nodule and has been proposed as an influential factor in predicting nodule malignancy, studies exploring the volume and mass of part‐solid nodules are unfortunately still scarce. Finally, the solid component has been demonstrated as an indicator of invasive tendency,^25^, ^26^ which is consistent with the values of the solid component in this study. Some recent studies have also insisted that the solid component is conducive to predicting invasiveness, compared with the monolithic component.^27^, ^28^, ^29^ For the CT setting, lung window imaging was used to measure the longest diameter of the solid component on the transverse section, referencing the revised guideline published in 2017. It has been argued persuasively that good agreement between radiologists, pulmonologists and observers and between residents and peers in measuring the solid component has been achieved.^14^ This provides a strong theoretical support for the measurement of the solid component. The critical value of the size of the solid component was calculated as 3.95 mm in this study. The corresponding sensitivity and specificity were 0.805 and 0.897, respectively. Previously, Lee et al. suggested that 3.0 mm [window width, 1500 HU; level, −700 HU] may be a safe and useful radiological standard for preoperative prediction of preinvasive and MIA lesions.^30^ Therefore, it can be expected that the predictive potential of these radiological results using AI and manual measurements will be better confirmed in future randomized prospective trials.

More interestingly, the threshold of the independent factors measured by AI mentioned above were consistent with or close to the threshold measured manually in previous studies. This finding lends credence to the idea that AI has the potential to identify malignant nodules. In addition, with the objective quantitative parameters of AI, radiologists cannot only reduce tedious manual work and avoid measurement error but also judge whether the solid elements of nodules change subtly through the increasing of CT attenuation value. This subtle change is impossible for radiologists to discover under the condition of certain lung window. As a result, the radiologist can pay more attention to the evaluation of the morphology of the nodules themselves and achieve an efficient comprehensive judgement.

This study has the following limitations. First, this was a retrospective study. Second, it is necessary to expand the sample to further verify the results. Due to the limited sample in the present study, the malignant part‐solid nodules were not classified into MIA and IA categories in this study, and the corresponding detailed parameter threshold was not calculated. In addition, CT images obtained with different scan parameters provided by different vendors may also have biased the diagnostic performance of AI. The AI in our study is a popular commercial deep learning model in our region, which is most commonly used in the detection of lung nodules and rarely used in judging malignancy. If the limitations of this study can be mitigated, it is expected that a more skillful deep learning model can be proposed in the future and that training and verification models can be established using multicentre data to illustrate the value of AI.

In conclusion, the accuracy of using AI to predict the nature of part‐solid nodules was reliable and comparable to that of senior radiologists in this study. The longest diameter of the whole nodule and the mean CT attenuation value of the parameters measured by AI, except for volume, were independent predictors for malignant part‐solid nodules. The longest diameter of the solid component measured by an experienced radiologist under the lung window was another independent factor for predicting malignancy of part‐solid nodules. Combined with the analysis of morphological characterization by senior radiologists, the authors preliminarily believe that the qualitative determination of part‐solid pulmonary nodules by AI can provide a certain value for the clinical management of patients in our region.

## AUTHOR CONTRIBUTIONS

Xiaoting Ke designed the study and wrote the manuscript; Weiyi Hu drafted the work; Xianyan Su and Fang Huang collected the data; Qingquan Lai analysed and interpreted data.

## CONFLICT OF INTEREST

The authors have no conflict of interest to declare.

## ETHICS STATEMENT

This study was approved by the Clinical Research Ethics Committee of the Second Affiliated Hospital of Fujian Medical University (Fujian, China) (275/2021). Written informed consent was obtained from all patients for their anonymized information to be stored in the hospital database and used for clinical research as well as to be published in this article.

## Data Availability

All data generated or analysed during this study are included in this published article (and its supplementary information files).
